# Outstanding fluoride removal from aqueous solution by a La-based adsorbent†

**DOI:** 10.1039/d2ra06284d

**Published:** 2022-10-26

**Authors:** Weisen Yang, Fengshuo Shi, Wenlong Jiang, Yuhuang Chen, Kaiyin Zhang, Shaoju Jian, Shaohua Jiang, Chunmei Zhang, Jiapeng Hu

**Affiliations:** Fujian Key Laboratory of Eco-Industrial Green Technology, College of Ecology and Resources Engineering, Wuyi University Wuyishan 354300 China jianshaoju@126.com wyuwqhjp@163.com; Jiangsu Co-Innovation Center of Efficient Processing and Utilization of Forest Resources, International Innovation Center for Forest Chemicals and Materials, College of Materials Science and Engineering, Nanjing Forestry University Nanjing 210037 China; College of Mechanical and Electrical Engineering, Wuyi University Wuyishan 354300 China; Institute of Materials Science and Devices, School of Materials Science and Engineering, Suzhou University of Science and Technology Suzhou 215009 China cmzhang@usts.edu.cn

## Abstract

A La-based adsorbent was prepared with La(NO_3_)_3_·6H_2_O, 2-methylimidazole and DMF *via* amide-hydrolysis and used for fluoride decontamination from aqueous water. The obtained adsorbent was lanthanum methanoate (La(COOH)_3_). The effects of pH value, initial F^−^ concentration and interfering ions on defluoridation properties of as-prepared La(COOH)_3_ were assessed through batch adsorption tests. The adsorption kinetics, isotherm models and thermodynamics were employed to verify the order, nature and feasibility of La(COOH)_3_ towards fluoride removal. The results imply that La(COOH)_3_ is preferable for defluoridation over a wide pH range of 2 to 9 without interference. Simultaneously, the defluoridation process of La(HCOO)_3_ accords to the pseudo-second order model and Langmuir isotherm, revealing chemical adsorption is the main control step. The maximum fluoride capture capacities of La(COOH)_3_ at 30, 40 and 50 °C are 245.02, 260.40 and 268.99 mg g^−1^, respectively. The mechanism for defluoridation by La(COOH)_3_ was revealed by PXRD and XPS. To summarize, the as-synthesized La based adsorbent could serve as a promising adsorbent for defluoridation from complex fluoride-rich water.

## Abstract 2

A La-based adsorbent was prepared with La(NO_3_)_3_·6H_2_O, 2-methylimidazole and DMF *via* amide-hydrolysis and used for fluoride decontamination from aqueous water.

## Graphical abstract

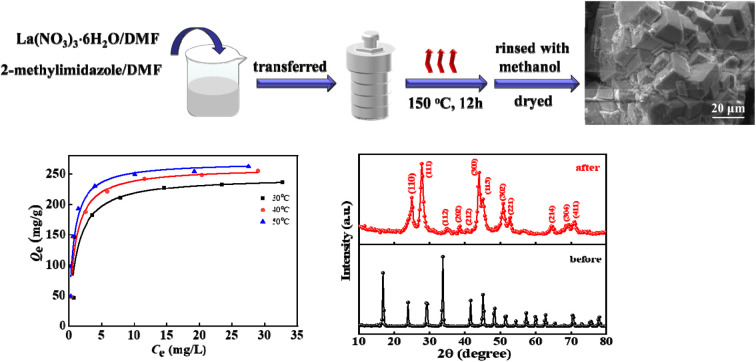

## 1. Introduction

The fluoride pollution in aquatic ecosystems has drawn global concern in recent years. Excessive intake of fluoride from water leads to thyroid disease, osteoporosis, skeletal and dental fluorosis, and even brain damage.^1^ Groundwater with high fluoride is exposed in more than 25 countries such as India, American, Pakistan, Africa, Sri Lanka, Mexico, China and so on.^2,3^ Accordingly, exploring an efficient and feasible fluoride remediation technology is imperative. Up to now, various remediation options for defluoridation such as anion exchange,^4^ membrane technology,^5^ adsorption,^6^ precipitation–coagulation,^7^ reverse osmosis (RO),^8^ and electrodialysis^9,10^ were conventionally established for fluoride retention. Among these water purification techniques, adsorption technology has been indicated to be an eye-catching and economical option owing to its comprehensive benefits of simplicity of design, operational flexibility, inexpensive expenditure, and high efficiency. A great variety of adsorbents like nano sized metal-oxide adsorbents,^11^ carbon-based adsorbents,^12^ glass fibers,^13^ montmorillonite,^2^ Mxenes,^14,15^ polymers,^16–18^ clay,^19,20^ and metal–organic frameworks (MOFs)^21–24^ have been designed to recover fluoride ions and metal ions in water. However, most traditional defluoridation adsorbents have the limitations of low removal efficiency, are highly pH-dependent over a narrow pH range or have poor selectivity.

Currently, adsorbents based on rare-earth metal element have been recognized as effective adsorbent materials. Zr MOFs based on ZrCl_4_ and tetrafluoroterephthalic acid exhibited good defluoridation performance over a wide pH range of 3 to 10 with the maximum uptake of 204.08 mg g^−1^ computed by Langmuir model.^25^ Various La-based materials have been wildly utilized for remediation of phosphate, metal ions, arsenic and fluoride because of the strong affinity and high selectivity of rare earth metal lanthanum (La), the biocompatible, the low cost, and environmentally friendly.^26–28^ The La-MOF@50%PANI, which was fabricated with terephthalic acid (1,4-BDC) ligand through a one-pot technique possessed superior removal efficiency toward Pb^2+^ with maximum capture uptake of 185.19 mg g^−1^.^29^ Yin *et al.* explored five La-MOFs *viz.* La-BTC, La-BDC, La-BPDC, La-PMA, and La-BHTA for immobilization of fluoride, the maximum uptake of fluoride reached 105.2, 171.7, 125.9, 158.9 and 145.5 mg g^−1^ at 25 °C, respectively.^3^ Fe–Mg–La tri-metal nanocomposite^30^ prepared by co-precipitation without calcination exhibit efficient defluoridation performance with maximum capacity of 47.2 mg g^−1^. Prabhu S M *et al.*^31^ developed La(HCOO)_3_ through an acid catalyst amide-hydrolysis mechanism using lanthanum nitrate hexahydrate, benzoic acid (BA) and DMF as materials. The maximum adsorption of AsO_4_^3−^ by La(COOH)_3_ was found to be 2.623 mmol g^−1^. Until now, La(COOH)_3_ prepared with La(NO_3_)_3_·6H_2_O, 2-methylimidazole and DMF *via* basic amide-hydrolysis mechanism and used as an adsorbent for defluoridation has never been reported.

Herein, La-based adsorbent (lanthanum methanoate) was fabricated *via* amide-hydrolysis mechanism for abating excess F^−^ from aqueous solution. Characterization of the as-prepared adsorbent was thoroughly evaluated by SEM, PXRD and XPS. The defluoridation property of La(COOH)_3_ was quantified in detail. To elucidate the mechanism of fluoride decontamination by La(COOH)_3_, the kinetics models and adsorption isotherms were systematically studied *via* adsorption.

## 2. Experimental

### 2.1. Materials

La(NO_3_)_3_·6H_2_O (99.9%), *N*,*N*-dimethylformamide (DMF, 99.5%), NaF (99%), 2-methylimidazole (98%), NaOH (≥98%), NaCl (99.5%), HCl (37%), CH_3_OH (99.9%), and other used chemicals were obtained from Aladdin Reagents Co., Ltd and used directly.

### 2.2. Synthesis of La-based adsorbent

**Scheme 1:**
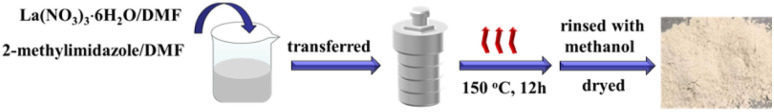
The diagram of the preparation of La-based adsorbent.

1.300 g of La(NO_3_)_3_·6H_2_O and 0.825 g of 2-methylimidazole were dissolved thoroughly in 40 mL of DMF under stirring for 10 min, respectively. Then the above solutions were mixed under vigorous stirring for 30 min, and reacted at 150 °C for 2 h in a 150 mL solvothermal autoclave. Subsequently, in order to eliminate the unreacted agents from pores of lanthanum methanoate, the precipitation was washed with reaction solvent and methanol several times and dried at 80 °C. The preparation scheme of La-based adsorbent was displayed in Scheme 1.

### 2.3. Adsorption tests

The 100 mg L^−1^ F^−^ standard stock solution was prepared by pouring an appropriate amount of NaF into 1 L deionized (DI) water. For batch defluoridation experiments, 0.01 g lanthanum methanoate was immersed in to 50 mL of F^−^ solution at 30 °C for 12 h in triplicate. Adsorption kinetics examinations were performed with 1 L of F^−^ solution (10 and 20 mg L^−1^ calculated by the mass of fluoride anions). To investigate the adsorption isotherms, the defluoridation experiments were conducted by varying the initial F^−^ concentration (10–80 mg L^−1^) at 30, 40 and 50 °C, respectively. The effect of pH adjusted by 0.1 M HCl or NaOH solution was examined in the range of 2–9 with 50 mL of 20 mg L^−1^ F^−^ solution. 10–50 mg L^−1^ of Ca^2+^, Mg^2+^, NO_3_^−^, Cl^−^, CO_3_^2−^, PO_4_^3−^ and SO_4_^2−^ were selected as interfering ions in adsorption condition (*C*_0_ = 10 mg L^−1^, pH = 8, *V* = 50 mL) to identify the selectivity of lanthanum methanoate. The residual F^−^ concentrations after adsorption were monitored by F^−^-selective electrode using the standard method,^2,32^ and the capture capacity of lanthanum methanoate was computed according to our previous work.^11^

### 2.4. Material characterization

The surface morphology and size of lanthanum methanoate were determined by scanning electron microscopy (VEGA300U, Tescan). The pristine and used lanthanum methanoate were verified by X-ray diffraction (D8 ADVANCE, Bruker) equipped with Cu Kα radiation in the 2*θ* range of 10–80° at 2° min^−1^. XPS measurements were examined using a Thermo Scientific K-Alpha spectrometer (Thermo Fisher, USA). A Nicolet 330 FTIR spectrometer (Thermo Fisher Scientific Ltd, USA) was used to record the Fourier transform infrared spectra (FTIR).

## 3. Results and discussion

### 3.1. Adsorbent characterization

**Fig. 1:**
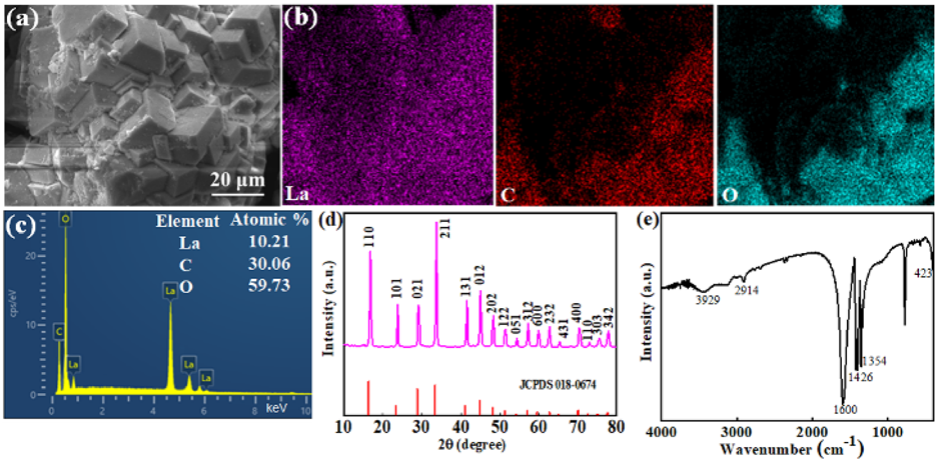
SEM micrograph (a), EDX elemental mapping (b), EDX analysis (c), PXRD (d) and FT-IR spectrum (e) of La-based adsorbent.

The surface morphology of La-based adsorbent is depicted in Fig. 1a. Clearly, it exhibits a strong polyhedron solid structure and aggregates in block with several small debris on the surface. The particle size of La-based adsorbent is micron with strong solid structure. Fig. 1b displays the EDX mapping of La-based adsorbent, the EDS spectrum indicates that the presence of C, O and La in the particles (Fig. 1c). The structure of the La-based adsorbent was verified by PXRD pattern and FTIR spectra (Fig. 1d and e). The La-based adsorbent possesses sharp and strong characteristic peaks at 2*θ* = 16.8, 23.8, 29.1, 33.8, 41.6, 44.9, 48.4, 51.5, 54.4, 57.3, 60.0, 62.8, 65.4, 68.1, 70.4, 73.0, 75.6 and 77.9°, which is corresponded to La(COOH)_3_ (JCPDS card No. 00-018-0674) reported in the literature^31,33,34^ (Fig. 1d), suggesting that the La-based adsorbent is pure La(HCOO)_3_. In the spectrum of La-based adsorbent (Fig. 1e), the band occurred at 3429 cm^−1^ corresponds to the –OH stretching vibration. The significant peaks appeared at 1600 cm^−1^ and 1426 cm^−1^ are indicative of C–O asymmetric and symmetric vibrations, revealing the carboxylic groups in the methanoate.^33^ The bands at 2914 cm^−1^ and 1354 cm^−1^ ascribed to the –CH stretching and bending mode illustrates the presence of methanoate,^31^ manifesting that the La^3+^ ions are successfully coordinated with the groups of –OOCH to generate the La(COOH)_3_. The peak located at 423 cm^−1^ represents the La–O stretching vibration in La(COOH)_3_.^33,34^ The La(COOH)_3_ may be generated *via* basic amide-hydrolysis mechanism and the possible pathway is illustrated in Scheme S1† in ESI.^35,36^

### 3.2. Effects of parameters on defluoridation performance

#### 3.2.1. Impact of the initial pH values on defluoridation

**Fig. 2:**
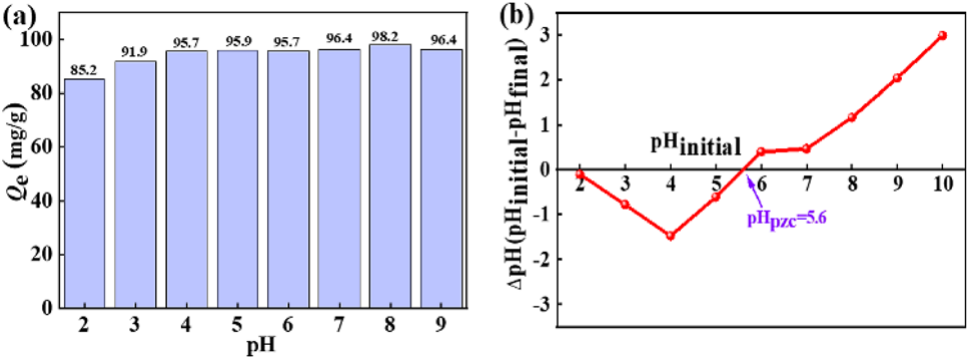
The impact of pH on defluoridation (a) and pH_pzc_ (b) of the adsorbent.

The change of pH value can not only affect the charge on the La(COOH)_3_ surface but also affects the existence species of fluoride in the solution. Fig. 2a illustrates the influence of pH on defluoridation of La(COOH)_3_. It is clear that La(COOH)_3_ exhibits an outstanding defluoridation efficiency over a very broad pH range of 2 to 9, the decontamination performance is more than 85 mg g^−1^ with original F^−^ concentration of 20 mg L^−1^ in this pH range. The highest uptake of fluoride is 98.2 mg g^−1^ at pH of 8. Even at pH of 9, the capture capacity of fluoride is 96.4 mg g^−1^. The removal efficiency (85.2 mg g^−1^) is relatively low at pH of 2, since part of F^−^ exist as HF at pH of 2.^1,37^ The isoelectric point (pH_p*z*c_) of La(COOH)_3_ assessed using pH drift method^38^ is noted as 5.6 (Fig. 2b), implying that the superficies of La(COOH)_3_ is positively charged when pH < 5.6 which is favourable for defluoridation, and negatively charged when pH > 5.6. Hence, the fluoride capture capacity maintains at a relative high level in the pH ranging from 3 to 6 because of the electrostatic attraction between the negative charged F^−^ and the positively charged La(COOH)_3_. La(COOH)_3_ is deprotonated when pH > pH_p*z*c_. However, with further increase of pH (7–9), the fluoride capture capacity is higher than 96 mg g^−1^ and reaches the highest value at pH 8, reflecting that the defluoridation of La(COOH)_3_ takes place not only by electrostatic attraction, but also through ligand exchange mechanism between the F^−^ and the –OOCH group of La(COOH)_3_ adsorbent as well as non-specific electrostatic attraction^3,37^ at alkaline medium. Hence, a pH of 8 is selected as the optimum pH for subsequent experiments.

#### 3.2.2. Impact of original F^−^ concentration

**Fig. 3:**
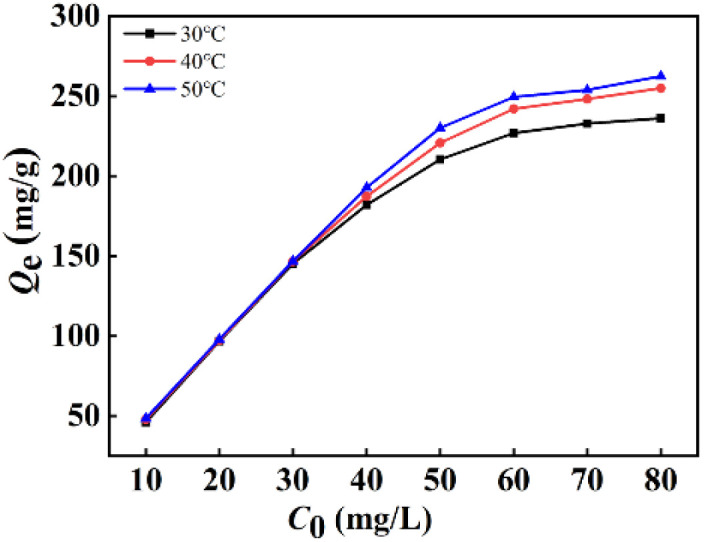
Impact of original F^−^ concentration on defluoridation by La(COOH)_3_.

Fig. 3 presents the impact of original F^−^ concentration in the range of 10 to 80 mg L^−1^ keeping adsorbent dosage fixed at 0.01 g on the defluoridation of La(COOH)_3_ at altered temperature for 12 h. Notably, the F^−^ capture capacity enlarges gradually with the rise of original F^−^ concentration range of 10 to 60 mg L^−1^ owing to the acceleration of the diffusion rate of F^−^ caused by concentration gradient, and then no significant variation in binding capacity is observed due to the equilibration of binding sites of the La(COOH)_3_ at higher-fluoride solutions under a constant dosage condition. Additionally, at a high fluoride concentration system, the defluoridation process of La(COOH)_3_ is favourable at higher temperature.

#### 3.2.3. Impact of interfering ions

**Fig. 4:**
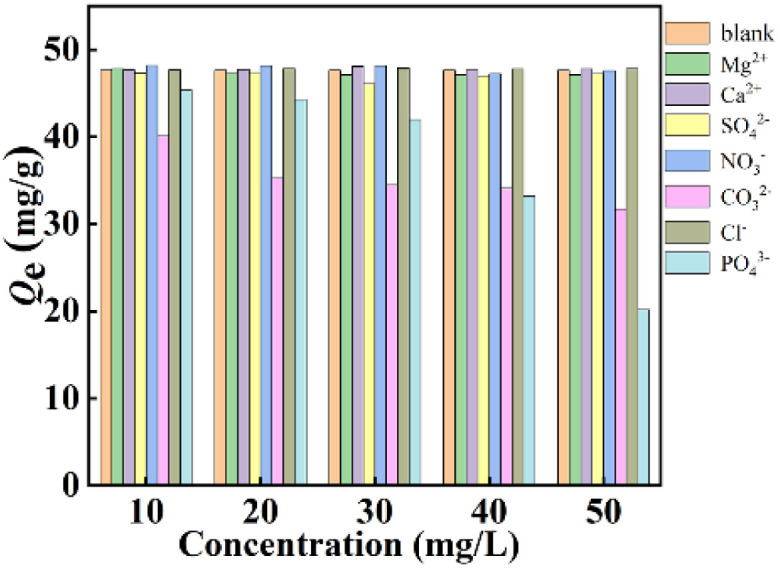
Effect of interfering ions on the defluoridation performance of La(COOH)_3_.

To evaluate different interfering ions efficiencies on the defluoridation process in the complex wastewater, Ca^2+^, Mg^2+^, NO_3_^−^, Cl^−^, CO_3_^2−^, PO_4_^3−^ and SO_4_^2−^ with different initial concentrations were poured into fluoride solution to form 50 mL of binary solutions containing 10 mg L^−1^ of F^−^ and various interfering ions concentrations (10–50 mg L^−1^), respectively. Clearly, the presence of interfering ions like Ca^2+^, Mg^2+^, NO_3_^−^, Cl^−^, and SO_4_^2−^ has little variety in defluoridation performance of La(COOH)_3_ (Fig. 4). Whereas PO_4_^3−^ and CO_3_^2−^ exhibit significant interference since the p*K*_sp_ of La(PO_4_)_3_ and p*K*_sp_ of La_2_(CO_3_)_3_ are 26.16 and 33.4,^39,40^ respectively, which are higher than that of LaF_3_ (18.5). The higher the concentration of PO_4_^3−^ and CO_3_^2−^ is, the greater interference intensity is. The binding capacities for F^−^ descend from 45.4 to 20.2 mg g^−1^ and 40.1 to 31.7 mg g^−1^ as the concentrations of PO_4_^3−^ and CO_3_^2−^ increase from 10 to 50 mg L^−1^, respectively. Taking all the above-mentioned factors into account, it can be concluded that La(COOH)_3_ prepared in this study has a significant anti-interference ability and the inhibitory effect of PO_4_^3−^ and CO_3_^2−^ should be particularly concerned in practical complex engineering application.

#### 3.2.4. Impact of contact time and adsorption kinetics

**Fig. 5:**
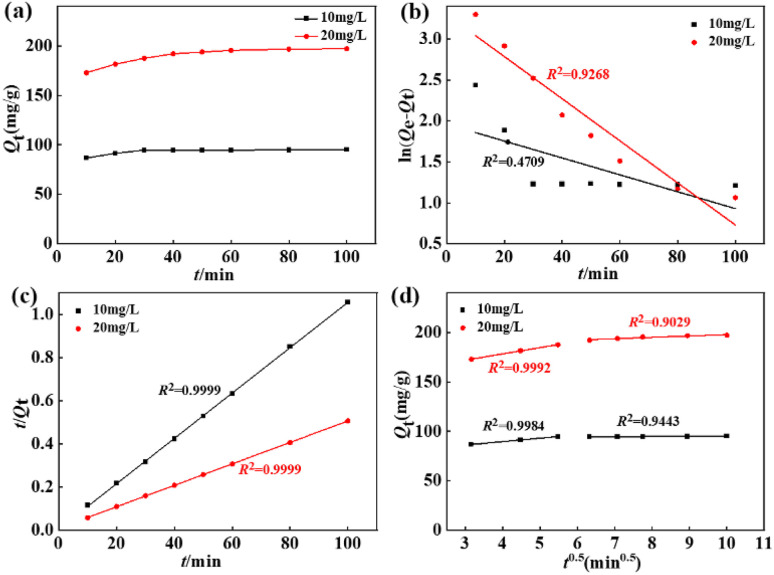
Effect of interfering ions on the defluoridation performance of La(COOH)_3_.

Fig. 5a depicts the time-dependent defluoridation on La(COOH)_3_ under the fixed adsorbent dose of 0.1 g and 1 L of F^−^ solution with various original F^−^ concentrations (10 and 20 mg L^−1^) at pH of 8, respectively. As seen from Fig. 5a, the binding capacity rises rapidly as time increases within 30 minutes, and then attains equilibrium at a contact time of 100 minutes. To interpret the rate-limiting step and determine the defluoridation behaviour of La(COOH)_3_, three commonly used reaction-based models^41^ were conducted to fit the experimentally measured data. The fitting patterns and dependable factors are recorded in Fig. 5b–d and Table 1. Notably, the pseudo-second-order model (PSO) with higher fitted determination coefficients (*i.e.*, 0.9999 at 10 mg L^−1^ and 0.9999 at 20 mg L^−1^) provide a better description for the kinetics dates than the pseudo-first-order model (PFO), revealing the strong chemical interaction. Meanwhile, the *Q*_e_ (*i.e.*, 95.15 and 201.21 mg g^−1^ at 10 and 20 mg L^−1^) of La(COOH)_3_ estimated using PSO are accorded with experimental ones (94.96 and 197.10 mg g^−1^ at 10 and 20 mg L^−1^, respectively). Additionally, the diffusion route of F^−^ in the adsorption system is determined by Weber–Morris diffusion model. As portrayed in Fig. 5d, two-platform stages observed in the intra-particle diffusion model mean that the defluoridation on La(COOH)_3_ is consist of multiple diffusion mechanisms. The initial fast stage takes place within 30 min, the maximum adsorption rates (*k*_id1_) at 10 and 20 mg L^−1^ F^−^ solutions are 3.4649 and 6.3507 mg g^−1^ min^−0.5^ during this period, while the adsorption rates (*k*_id2_) in the subordinate stage occurred between 30 and 100 minutes decline to 0.0964 and 1.3654 mg g^−1^ min^−0.5^, respectively. Notably, none of the two-stage fitting line segments pass through the origin point, which illustrates that the defluoridation process by La(COOH)_3_ is governed by intra-particle diffusion as well as affected by diverse diffusion mechanisms involving surface adsorption, intra-pore diffusion, and external mass transfer.

**Table 1:** The fitted factors of kinetics models for defluoridation on La(COOH)_3_

Model	Parameter	*C*_0_ (mg L^−1^)	
		10	20
PFO	*k*_1_ × 10^2^ (min^−1^)	1.0320	2.5710
	*Q*_e_ (mg g^−1^)	7.09	27.11
	*R* ^2^	0.4709	0.9268
PSO	*k*_2_ × 10^2^ (g mg^−1^ min^−1^)	1.9108	0.2561
	*Q*_e_ (mg g^−1^)	95.15	201.21
	*R* ^2^	0.9999	0.9999
Weber–Morris	*k*_id1_ (mg g^−1^ min^−0.5^)	3.4649	6.3507
	*R* ^2^	0.9984	0.9992
	*k*_id2_ (mg g^−1^ min^−0.5^)	0.0964	1.3654
	*R* ^2^	0.9443	0.9029

#### 3.2.5. Adsorption isotherm

**Fig. 6:**
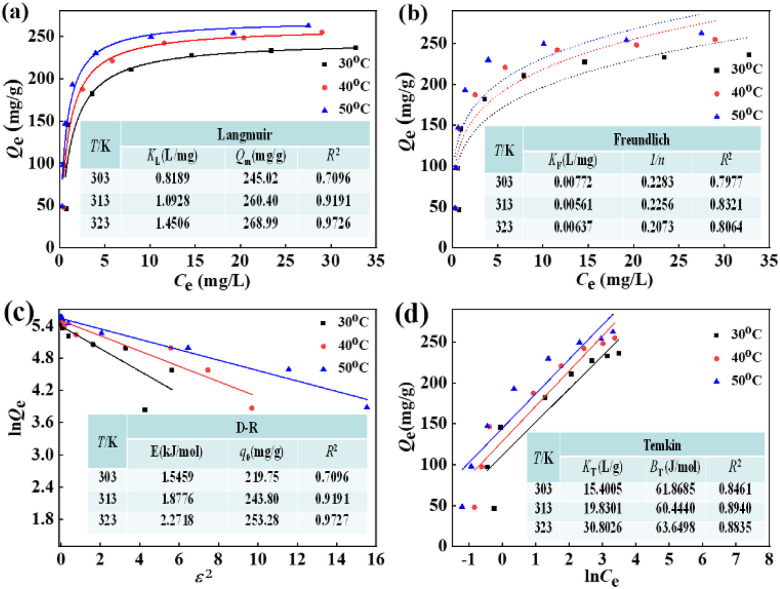
Adsorption isotherms of F^−^ on La(COOH)_3_ (a) Langmuir, (b) Freundlich, (c) D–R and (d) Temkin under various temperatures (30–50 °C).

Four classical adsorption isotherms including D–R, Langmuir, Temkin, and Freundlich^42–44^ were applied to identify the interactions between the F^−^ equilibrium concentration and binding uptake by La(COOH)_3_ at altered temperature. As displayed in Fig. 6a–d, the corresponding regression coefficients (*R*^2^) at temperatures of 30–50 °C are as follows: Langmuir (0.9019, 0.9367 and 0.9644) > Temkin (0.8461, 0.8940 and 0.8835) > Freundlich (0.7977, 0.8321 and 0.8064) > D–R (0.6908, 0.7685 and 0.7753), signifying that the single-layer and homogeneous adsorbed on La(COOH)_3_ dominates the process of defluoridation on the adsorbent. The maximum uptakes for Langmuir models are 245.02, 260.04 and 268.99 mg g^−1^ at 30, 40 and 50 °C, respectively, which are quite close to experimental values. Obviously, they are much higher than those of reported similar adsorbents listed in Table 2. The free energy (*E*) is computed using the D–R model, as depicted in Fig. 6c. From the calculations, the *E* values of La(COOH)_3_ for fluoride adsorption are 13.3492, 13.8554 and 14.6897 kJ mol^−1^ at 30–50 °C, respectively. It is evident that the values of *E* from 8 to 16 kJ mol^−1^ manifest that both ligand exchange and electrostatic attraction are the mainly mechanism.^3,43,45–47^ To portray the affinity between the F^−^ and the La(COOH)_3_, the dimensionless constant separation factor (*R*_L_) values calculated according to the literature^21,37^ decline quickly with the increasing of initial F^−^ concentrations in the range of 10–80 mg L^−1^ and approach to zero, manifesting that the adsorbing system is encouraged at even higher F^−^ concentrations (Fig. S1†). In addition, the values of *R*_L_ drop as temperature rises and are less than 1. Hence, the defluoridation process of La(COOH)_3_ occurs under favorable condition.

**Table 2:** Comparison of defluoridation capacity for La(COOH)_3_ and other studied adsorbents

Adsorbents	pH	*C*_0_ (mg L^−1^)	*T*/°C	*Q*_m_ (mg g^−1^)	Ref.
HAp-La based MOF	6	8–14	30	4.25	46
La-BDC	5	10–100	25	171.7	3
Ce-TDC MOF	3–9	5–100	25	94.9	45
Al-BTC MOFs	3–10	5–50	25	31.69	48
La@Fu MOF	7	8–14	30	4.75	43
Zirconium based MOFs	3–9	50–700	30	204.08	1
Lanthanide-based MOFs	3–7	—	25	103.95	49
MOF-801	—	8–256	100	166.11	50
MCH-La	3–11	0–80	25	136.78	51
La_2_O_3_–CeO_2_–Fe_3_O_4_	2–10	10–60	25	216.45	52
Bx-Ce-La@500	1–13	10–50	25	88.13	53
Fe–Mg–La	7	10–300	35	47.20	30
La(COOH)_3_	2–9	10–80	30	245.02	This study
			40	260.04	
			50	268.99	

#### 3.2.6. Thermodynamics studies

To explore the effect of temperature on the defluoridation of La(COOH)_3_, the thermodynamic investigation is utilized to study the defluoridation process (Fig. S2† and Table 3). The calculated Δ*H*^0^ (41.41 kJ mol^−1^) and Δ*S*^0^ (166.28 J mol^−1^ K^−1^) indicate that the fluoride removal onto La(COOH)_3_ is an endothermic nature and random state, revealing that the increase of temperature promotes the capture of fluoride. Δ*G*^0^ values are −10.26, −12.24 and −13.66 kJ mol^−1^ at 30–50 °C, respectively, demonstrating that the defluoridation process on La(COOH)_3_ is highly feasible and spontaneous. In addition, the defluoridation mechanism can be further elucidated by thermodynamic factors. The calculated Δ*H*^0^ (41.41 kJ mol^−1^) is larger than physical adsorption (2.1–20.9 kJ mol^−1^), but less than predicted chemical adsorption (80–400 kJ mol^−1^), implying that the defluoridation on La(COOH)_3_ is composed of chemisorption and physisorption process.

**Table 3:** Thermodynamic factors of defluoridation on La(COOH)_3_

*T* (°C)	Δ*G*^0^ (kJ mol^−1^)	ln *K*_D_	Δ*H*^0^ (kJ mol^−1^)	Δ*S*^0^ (J mol^−1^ K^−1^)
30	−10.26	58.63	41.41	166.28
40	−12.24	110.30		
50	−13.66	162.86		

### 3.3. Adsorption mechanism *via* PXRD and XPS characterization

**Fig. 7:**
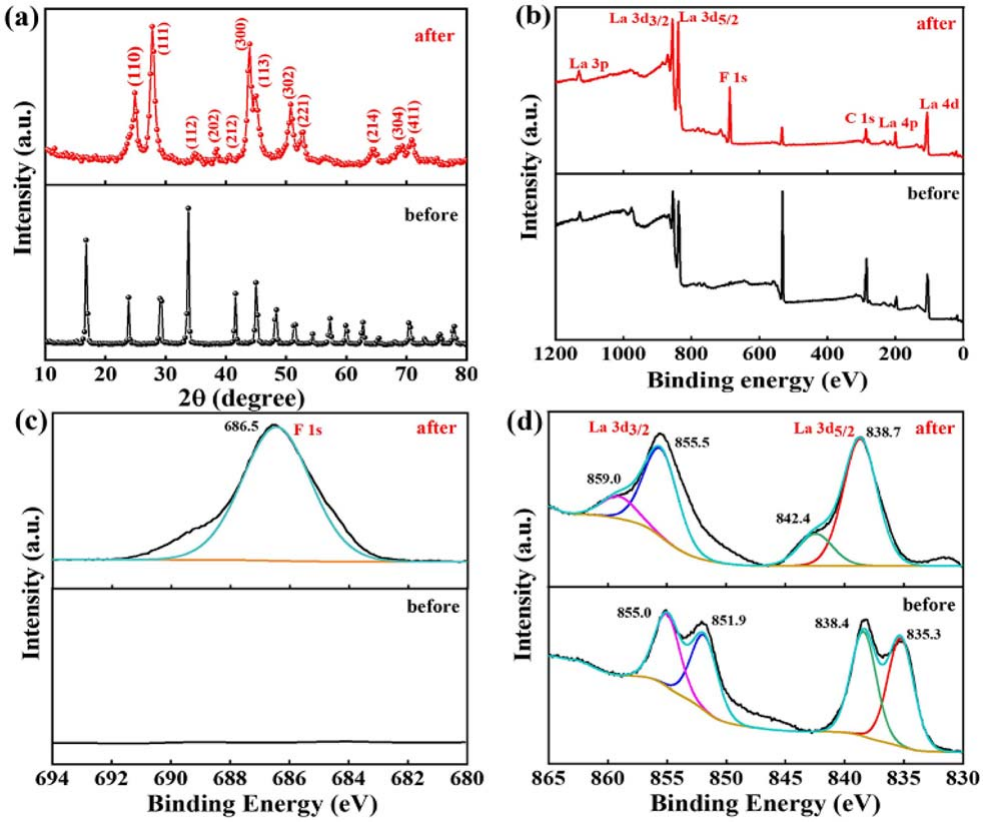
The used La(COOH)_3_ PXRD patterns (a), the fresh and used La(COOH)_3_ XPS analysis: (b) survey spectra, (c) F 1s and (d) La 3d of La(COOH)_3_.

To gain deeper insight into the mechanism of defluoridation by La(COOH)_3_, PXRD and XPS analysis is recorded to verify the changes in structure of pristine and used adsorbent. After fluoride uptake, the characteristic peaks belonged to La(COOH)_3_ disappear and a series of new reflections corresponded to the hexagonal phase of LaF_3_ (PDF No. 32-0483) emerge in the PXRD pattern^52,54^ (Fig. 7a), signifying that precipitation and ligand exchange is identified as the dominating defluoridation mechanism.

**Scheme 2:**
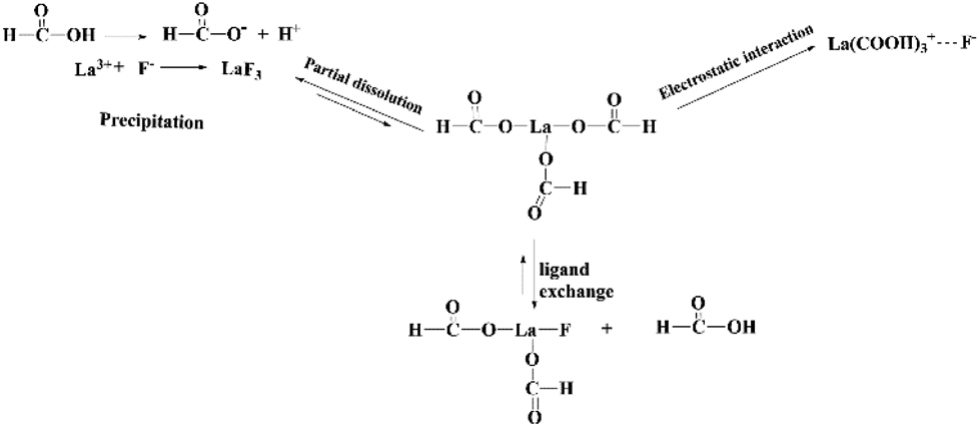
Possible mechanism of defluoridation using La(COOH)_3_.

Furthermore, XPS analysis is conducted to further elucidate the structural composition of the fresh and used adsorbent. As depicted in Fig. 7b, obvious signals of C and La can be found in the survey spectrum of fresh La(COOH)_3_. As for La 3d, two peaks appeared at 835.3 and 838.4 eV are corresponded to La 3d_5/2_ spin state, while peaks located at 851.9 and 855.0 eV are ascribed to La 3d_3/2_ orbital.^31,55^ After defluoridation, a new F 1s peak centred at 686.5 eV with a slight shift (∼1.8 eV) to high binding energy compared with the F 1s standard spectrum of NaF (684.7 eV) emerges in the spectrum,^56,57^ revealing that fluoride binding to the La(COOH)_3_ (Fig. 7c). Meanwhile, the La 3d_5/2_ peaks detected at 838.7 and 842.4 eV, and La 3d_3/2_ peaks appeared at 855.5 and 859.0 eV shift to high binding energy direction (3.4–4.0 eV), is attributed to the bond of La–F formed *via* ion exchange as well as precipitation^3^ (Fig. 7d). Hence, according to the isoelectric point (pH_p*z*c_) of La(COOH)_3_, PXRD and XPS analysis, the defluoridation process onto La(COOH)_3_ is significantly controlled by precipitation, ligand exchange between La and fluoride along with the electrostatic interaction, which is in agreement with the previous results obtained from kinetic and isotherm analysis. The possible defluoridation mechanism is shown in Scheme 2.

## 4. Conclusions

Lanthanum methanoate with particle size in micrometre was successfully fabricated for abating excess F^−^ from aqueous solution. La(COOH)_3_ functioned excellently over a wide pH variety from 2 to 9, with the largest decontamination performance at pH of 8. Fluoride adsorption behaviour on La(COOH)_3_ conform with the PSO model and Langmuir isotherm well, reflecting that single-layer chemisorption. The maximum uptakes of La(COOH)_3_ for fluoride achieve 245.02–268.99 mg g^−1^ at the temperatures of 30–50 °C, respectively, which are better than those of most adsorbents based on rare earth metal elements recorded in the literature. The defluoridation mechanism of La(COOH)_3_ is presided by precipitation, ligand exchange as well as electrostatic attraction. The results illustrate that the synthesized La-based adsorbent can be developed to immobilize fluoride-rich water.

## Author contributions

Conceptualization, investigation, writing – original draft preparation, Weisen Yang, Fengshuo Shi and Shaoju Jian; analysis, Wenlong Jiang; data curation, Yuhuang Chen; methodology, Kaiyin Zhang; writing – review & editing, Shaohua Jiang and Chunmei Zhang; project administration and supervision, Jiapeng Hu. All authors have read and agreed to the published version of the manuscript.

## Conflicts of interest

There are no conflicts to declare.

## Supplementary Material
